# Protocols versus practice: unravelling clinical checking variations in community pharmacies in England—a multi-method study

**DOI:** 10.1007/s11096-024-01743-9

**Published:** 2024-06-01

**Authors:** Ali Elgebli, Jason Hall, Denham L. Phipps

**Affiliations:** 1 Division of Pharmacy and Optometry, Faculty of Biology, Medicine and Health, The University of Manchester, Room 1.132, Stopford Building, Oxford Road, Manchester, M13 9PL UK; 2 Division of Pharmacy and Optometry, Faculty of Biology, Medicine and Health, The University of Manchester, Room 1.183, Stopford Building, Oxford Road, Manchester, M13 9PT UK; 3 Division of Pharmacy and Optometry, Faculty of Biology, Medicine and Health, The University of Manchester, Room 1.34, Stopford Building, Oxford Road, Manchester, M13 9PT UK

**Keywords:** Adherence to protocols, Clinical checking, Community pharmacy, Standards operating procedures (SOPs)

## Abstract

**Background:**

Standardisation, a widely accepted concept for risk management, entails designing and implementing task-specific operating procedures. In community pharmacies, Standardised Operating Procedures (SOPs) are a mandatory requirement and are recognised as essential for upholding safety and quality.

**Aim:**

This study aimed to investigate community pharmacists’ (CPs) compliance with SOPs when checking prescriptions, and the reasons for variations between standardised protocols and practice.

**Method:**

Eight sets of SOPs underwent hierarchical task analysis (HTA) to generate a normative description of clinical checking execution as per protocols. Subsequently, twelve CPs were engaged in a simulated clinical checking exercise, verbalising their thoughts while checking virtual prescriptions. Transcribed data underwent content analysis, aligned with a descriptive model to uncover engagement patterns, and disparities between SOPs and CPs’ practices. Finally, a focus group discussion took place to contextualise the observed variations.

**Results:**

HTA aided in constructing a clinical checking model with six primary subtasks and 28 lower subtasks. CPs often omitted subtasks during checks, diverging from prescribed protocols. These deviations, observed in controlled environment, reveal an ingrained aspect within the professional culture of pharmacists, where there may be a tendency not to strictly adhere to protocols, despite variations in work conditions. Contributing factors to this culture include the exercise of professional judgment, reliance on others, and prioritisation of patient preferences.

**Conclusion:**

This study highlights ongoing deviations from SOPs during clinical prescription checks in community pharmacies, suggesting a cultural tendency. Future research should delve into risk management strategies for these deviations and address the delicate balance between flexibility and stringent compliance.

**Supplementary Information:**

The online version contains supplementary material available at 10.1007/s11096-024-01743-9.

## Impact statements

- Pharmacists deviate from SOPs during clinical checks, and these deviations were observed in controlled, quiet conditions, suggesting a cultural inclination to depart from standard procedures despite the absence of external influences.
- The practice of diverging from clinical checking SOPs is thought to be rooted in the exercise of professional judgment, reliance on external resources, and prioritisation of patient preferences, particularly efficiency.
- Developing policy initiatives is needed to mitigate the potential risks associated with deviations from clinical checking SOPs and establish methodologies that balance operational efficiency with compliance to established protocols.

## Introduction

Standardisation is a familiar and often-advocated risk management concept in healthcare [1]. It is achieved through designing and implementing a set of procedures which describe how a particular task should be performed [1, 2]. In healthcare, the shift towards standardisation of task performance has been established to mitigate errors through the introduction of systematic procedures and protocols [1]. Since 2005, UK community pharmacies have been under a mandate to adopt Standardised Operating Procedures (SOPs), compelling each pharmacy to maintain current SOPs that outline the processes for routine tasks, with regulatory and professional obligation to follow these procedures [3]. Such regulations were introduced to ensure the minimum safety standards are met, and to promote consistent adherence to best practices [2, 4].

The introduction of SOPs serves to offer reassurance and establish a safety threshold, safeguarding patients from potential harm [2]. However, the success of these procedures is contingent upon the staff's strict adherence to them. There has been debate over how realistic or beneficial it is to enforce such strict compliance. Some argue that while these procedures are meant to safeguard patient care, they can sometimes be unrealistic or overly restrictive, which may influence the autonomy and flexibility of healthcare professionals [5, 6].

In pharmacies, SOPs manifest as a collection of protocols that employees are expected to comply with to maintain a high standard of consistency and service quality; they include a set of rules, regulations, laws, or established procedures [3]. That being said, emerging evidence from different healthcare settings [7, 8], including community pharmacies [9], suggests that employees often depart from SOPs in their day-to-day work. These deviations have been attributed in the literature [10, 11] to factors such as; the exercise of professional judgment (sometimes conflicting with established protocols); the drive for efficiency; the need to overcome workplace challenges; and constraints in resources that hinder strict compliance.

Community pharmacy is a unique and complex socio-technical system, existing both as a commercial business and as a provider of publicly-funded healthcare services [12]. Ashour et al. [11] and Thomas et al. [6] have identified several instances of pharmacy staff working outside of established protocols. In turn, SOPs have been criticised for oversimplifying tasks and for their limited ability to account for environmental factors like staffing levels, workplace pressures, and interruptions, all of which are believed to influence how tasks are executed in real-world practice [6]. Whilst diverging from the protocols may improve efficiency in practice [11], they can also give rise to safety concerns regarding the balance between flexibility and compliance. Also, deviating from SOPs raises concerns regarding whether SOPs are effectively fulfilling their intended objectives of ensuring consistency, quality, and safety [3].

In this study, we examine CPs’ compliance with SOPs during clinical checking, specifically CPs practising in England [13]. This is considered to be a particularly critical and skilled part of the dispensing process; so much so that, unlike other dispensing tasks, it cannot be delegated to anybody other than the pharmacist. It also relies heavily on professional judgment [14], which has been cited as a factor leading to deviation from SOPs [6]. Hence, understanding the extent to which CPs follow SOPs when checking prescriptions is important, particularly in the light of recent findings which revealed pronounced variations in how CPs define and conduct clinical checks [15].

### Aim

This study aimed to examine CPs' compliance with SOPs during clinical checking and the reasons why any deviations might occur.

### Ethics approval

The interviews that constituted the simulation task in this study received ethical approval from the University of Manchester Ethics Committee (Reference 2021–13400), whereas an ethical exemption was granted for the focus group.

## Method

### Overview

The study began with creating a normative description of clinical checking through analysing SOPs. This normative description served as a performance benchmark. Next, CPs’ checking behaviour from a simulated checking exercise was compared against the normative description to identify deviations. Additionally, a focus group discussion with senior CPs contextualised the observed variations. The dataset was managed using NVIVO [16], and Bing AI technology was utilised strictly to provide language guidance for the initial draft, which went through rounds of revisions by the authoring team.

### SOPs, materials and analysis

**Fig. 1:**
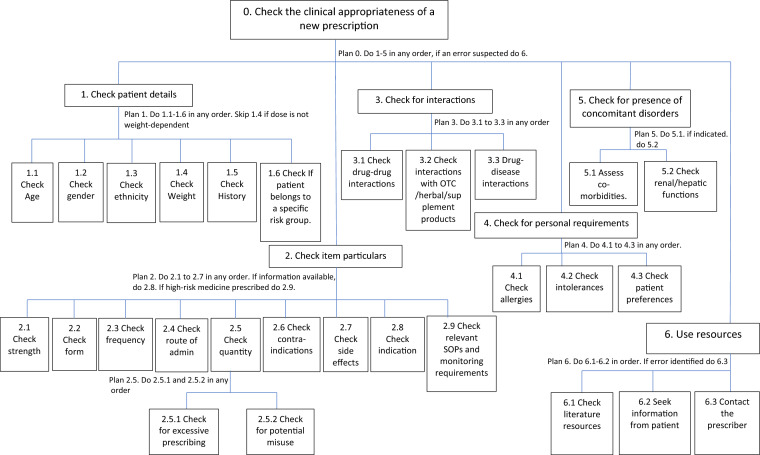
HTA of the standard operating procedures

We used a Hierarchical Task Analysis (HTA) [17] to examine a collection of SOPs gathered from community pharmacies and professional organisations. HTA has seen prior application within healthcare [11, 18], showcasing adeptness in handling intricate tasks, hence its adoption, in this study, to create a descriptive model of clinical checking. We collated a total of eight SOP documents, representing nearly all community pharmacy models. The clinical checking SOPs exhibited high similarity. Specifically, those obtained from independent pharmacies were identical, aligning with the SOPs published by the Pharmacy Defence Association [19]. The analytical approach adopted in this study involved an iterative process of reviewing SOPs while conforming to Santon's [17] prescribed methodology for conducting a HTA. The model derived from this process (Fig. 1) comprised of six first-level sub-tasks, twenty-eight lower-level sub-tasks and eight plans.

The model was initially developed by AE, a community pharmacist, and a researcher, then underwent a review by DP, an ergonomics and human factors specialist, and JH, an Academic pharmacist. Subsequently, refinement occurred through a panel discussion conducted at a university premises involving five expert community pharmacists, including managers, superintendents, and pharmacy owners. These professionals, recruited from a community pharmacy collaborative research team affiliated with the authors' institution. During the hour-long discussion, participants reviewed the initial model to ensure its alignment with SOPs and collectively approved a final version representing SOP-compliant task execution. Due to their knowledge of study materials, these group members were not eligible to participate in the simulation exercise.

### Clinical checking simulation exercise

Twelve CPs (Table 1) were recruited using purposive sampling [20] from a sampling frame comprising CPs working in England. Potential participants were approached through social media posts and the professional networks. Those eligible to participate included practicing CPs registered with the General Pharmaceutical Council and experienced in clinical checking.

**Table 1:** Characteristics of the community pharmacists participating in the study

Pharmacist	Gender	Years of experience in community pharmacy	Nature of work	Organisation
1	Male	One	Employed	Large multiple
2	Male	Two	Employed	Independent store
3	Male	Two	Employed	Independent chain
4	Female	Six	Employed—chain lead pharmacist	Independent chain
5	Female	Seven	Locum	N/A
6	Female	Five	Locum	N/A
7	Female	One	Employed	Independent chain
8	Male	Two	Employed	Small multiple
9	Male	One	Employed	Large multiple
10	Male	Twenty	Owner—store manager	Independent pharmacy store
11	Male	Four	Employed—branch manager	Independent chain
12	Female	Two	Locum	N/A

Participants, interviewed between April and August 2022, were tasked with clinically checking three simulated prescriptions (supplementary materials) during online interviews. The prescriptions were crafted to encompass medications carrying different risk levels. This process was guided by a literature search, providing insights into the risk levels associated with medicines, including those linked to higher or lower rates of complications and hospitalisations. As a result, three prescription scenarios were developed, each featuring medications with differing risk profiles and severity levels. Moreover, patient cohorts were designed to encompass diverse patients’ groups, such as paediatrics, geriatrics, and pregnancy, while also ensuring the relevance of medical history associated with each prescription. The prescriptions underwent evaluation by a clinical pharmacist and a panel of CPs, affirming their efficacy in providing detailed insights into the checking task.

A concurrent think-aloud technique [21] was employed during the simulation for data collection. Participants verbalised their thoughts out-loud during this process, which was audio-recorded and transcribed verbatim by a university-approved transcriber. In this paper, we primarily examined pharmacists' compliance with SOPs and the specific sub-tasks they undertake during prescription checks. A comprehensive analysis of CPs' decision-making processes during this checking exercise, along with an evaluation of their decisions, is detailed in a separate paper.

### Comparison of CPs' clinical checking against SOPs

Data obtained from the simulation underwent deductive content analysis, guided by the clinical checking descriptive model (Fig. 1). This method involved a systematic comparison of participants' work process during the simulation with the respective sub-tasks and plans outlined within the model. The model served as the coding scheme, facilitating the systematic categorisation of data and the assignment of numerical codes to qualitative elements. This utilisation of content analysis enabled the transformation of qualitative data into numerical values. The analysis process was carried out using NVIVO, which facilitated the categorisation of responses and the subsequent calculation and computation of *checked* frequencies for each sub-task (Table 2).

**Table 2:** Number of times each sub-task was checked by the participant (N = 12)

First-level sub-tasks	Associated lower-level sub-tasks	Prescription 1	Prescription 2	Prescription 3
		No. of times checked	No. of times checked	No. of times checked
Patient details	Risk group	6	12	11
	Weight	0	0	5
	Ethnicity	0	0	0
	History	11	10	9
	Gender	7	12	12
	Age	11	12	12
	Indication*	3	10	9
Personal requirements	Allergies	0	0	9
	Preferences	0	0	11
	Intolerances	0	0	0
Concomitant disorders	Renal/hepatic function	2	N/A	N/A
	Assess co-morbidities	6	3	8

	Each prescribed item	Naproxen	Paracetamol	Labetalol	Aspirin	Amoxicillin	Prednisolone	Spacer device
Items particulars	Strength	9	3	10	10	12	10	N/A
	Form	9	3	9	5	10	9	3**
	Frequency	10	6	10	10	10	9	N/A
	Side effects	3	0	3	0	2	0	N/A
	Quantity	6	0	0	0	6	7	10
	Contraindications	11	1	4	1	4	0	N/A
Interactions	Drug-drug interaction	9	2	7	3	2	0	N/A
	Drug-disease interaction	2	0	0	2	0	0	N/A
	Interaction with OTC-herbal/supplements	1	1	0	0	0	0	N/A

Notes: ^*^Indication was not included in the prescriptions, yet interviewees tended to frequently consider during clinical checking. The simulated prescriptions did not include indications to resemble real practice as the vast majority of prescriptions in practice do not have a specified indication. All SOPs included in the analysis encourage pharmacists to check the indication if available. In this simulation study, pharmacists consistently accounted for the potential indication even when it was not explicitly stated in the prescription

^**^In this context, the term 'form' pertains to the evaluators assessing the suitability of the inhaler device. Specifically, only three participants recognised the unsuitability of the inhaler device, prescribed for a child despite being an adult device

### Focus group

To explain any differences found between the way that SOPs prescribe clinical checking and the way that CPs conducted the task, we carried out a focus group involving expert CPs (average experience > 10 years). The focus group members were experienced in discussing safety concerns that arise in community settings. They were recruited from a community pharmacy collaborative research team affiliated with the author's institution. The group discussion was held at the university's facility in March 2023.

Prior to the focus group discussion, participants were provided with the descriptive model (illustrated in Fig. 1), an overview of CPs' deviations from SOPs documented in Table 2, and the simulated prescriptions. During the session, participants were encouraged to provide their insights and opinions regarding the underlying factors contributing to the observed variations between SOPs and CPs’ checking. The discussion was recorded, transcribed verbatim, and subjected to inductive thematic analysis, following the methodology outlined by Braun et al., [22]. All research team members were involved in the analysis of SOP documents, simulated task, and focus group, with AE initially conducting the analysis and later deliberating upon with DP and JH.

## Results

### Hierarchical task analysis

Figure 1 shows the output of the HTA for clinical checking, based on the SOPs.

### Observed variations between CPs' checking and SOPs

As illustrated in Table 2, participants displayed deviations from SOPs across various instances, with their compliance varying both between different scenarios and within similar prescriptions. This observation suggests, not only deviations from protocols, but also a differential scrutiny of specific drugs and subtasks, despite their equal requirement for inspection under SOPs. To ascertain the significance of these disparities, a chi-square test was conducted with a significance level of 5% and 40 degrees of freedom. The test yielded a chi-square statistic of 61.44, exceeding the critical value of 55.76, with a resulting p-value of 1.62%, indicating statistical significance (χ^2^(40) = 61.44, p = 0.0162).

### Focus group analysis

#### Patients’ factors

The desire to achieve favourable outcomes for patients was proposed as a motivation to diverge from protocols. This highlights a potential conflict between prioritising safety and efficiency, as CPs sometimes omitted sub-tasks to meet patients' expectations. This conflict is thought to impact the extent to which prescriptions are meticulously checked against the SOPs.

*“It [following protocols strictly every time] is safer but less efficient probably”. [Participant 2, manager (medium chain)]*

Indeed, it was suggested that patients tend to prioritise the efficiency of services over their safety.

*“The outcome for the patient is very rarely safety; it’s efficiency and speed. If a pharmacist, if their checking time was to go up and prescriptions were to take longer, then the one outcome the patient would be bothered about is how quickly they’re getting their prescription”. [Participant 3, manager (large multiple chain)].*

Furthermore, compliance with SOPs was likely to vary when checking a prescription for regular patients compared to those presenting for the first time. Typically, more sub-tasks were involved when assessing a prescription for a regular patient because of the access to their pharmacy record, familiarity with their conditions. In contrast, when evaluating an acute prescription for a walk-in patient, there may not be enough information available to check with the same level of detail.

*“If they are not your regular patients you will not have the record of PMR so you would have to check the SCR [summary care records] and for that you need permission, consent and that might put some pharmacists off checking. [Participant 4, manager (Large multiple chain)]*

#### Professional judgement

It was also noted that pharmacists' professional judgment played a major role in determining the significance of various sub-tasks and, consequently, compliance with SOPs. Pharmacists typically conducted an initial screening of item particulars, which informed their judgment regarding which sub-tasks were pertinent to check.

*“I’d say … I think about an initial scan, and then you’re going back, right what I am dealing with here […] looking for the first item, you might clock then that there’s some labetalol on there and that kind of aspirin […] and two tablets once a day, I only ever really see that in pregnancy”. [Participant 1, Superintendent (independent pharmacy)].*

Experience was discussed as a contributory factor to the “initial scan” when judging which sub-tasks were relevant in a given scenario. Through recurrent encounters with similar scenarios, CPs developed a sense of which aspects of SOPs were most critical and concentrated on those. The pharmacists appreciated this behaviour did not guarantee that all the important sub-tasks were accounted for, but it worked well in optimising safety and efficiency.

*“Through clinical judgement and experience you’ll realise actually the most important thing is to check these 5 things or 4 things or whatever it is so I think generally it does not affect patient safety but there is obviously a risk that you could miss something”. [Participant 4, manager (large multiple chain)].*

#### Work conditions

Unsurprisingly, the stressors induced by the workplace such as time shortage, workload, multi-tasking, and lack of information were believed to impact compliance. In this study, the checking exercise was completed in a controlled environment, thus not providing an insight into the effects of environmental factors on SOP adherence. However, the focus group participants emphasised that, in practice, pharmacists would omit some sub-tasks due to the demanding workload.

*“If you came out of the consultation room with five baskets waiting, the shop’s full of people and the phone’s ringing, and the first basket there’s two boxes of naproxen and a paracetamol it would go straight out, and you’d just say, take them with food, and then you’d go to the next one”. [Participant 2, manager (medium chain)]*

The lack of information was identified as a barrier not only to protocol compliance but to the overall quality of the check. In cases where information on subtasks such as indication, renal, and liver functions are unavailable, CPs may resort to a "get-out clause," rather than deviating from protocols outright, rendering them unable to verify those parameters. Despite this, CPs may still consider such parameters, as shown in Table 2, even when not explicitly stated on a prescription. The SOPs typically include the phrase "check if available", which panel members believe is intended for "best-case scenarios" and to protect the organisation rather than expecting pharmacists to verify them routinely.

*“Most of the time you wouldn’t check renal or hepatic function because you wouldn’t have access to that information. The best you could do is ask someone if they’ve got any”. [Participant 1, Superintendent (independent pharmacy)]*

*It [including checking for renal/hepatic functions in SOPs] is done to cover the company because then if there was an incident the superintendent would have to prove that they had done everything to facilitate their pharmacists to do their job. [Participant 3, manager (large multiple chain)]*

#### Relying on others

In practice, CPs tended to adopt a reactive rather than proactive approach to certain sub-tasks, addressing them primarily in response to patient feedback. For instance, tasks like checking for intolerances, allergies, side effects, or interactions with OTC products often require active patient cooperation.

*“I wouldn’t actively check. I wouldn’t go out and ask a patient what you are taking over the counter […] I wouldn’t tend to, like a citalopram I wouldn’t go out and say, oh are you taking some St John’s Wort […] and often when you give the prescription out to the patient the patient might ask a question which may prompt you”. [Participant 3, manager (large multiple chain)]*

Also, pharmacists relied on prompts generated by the dispensing software to help them prioritise and check certain sub-tasks.

*“I think with this one probably I would be relying on it coming up on the proscript, [A dispensing system] because I feel like it would flag up on naproxen and citalopram”. [Participant 1, Superintendent (Independent pharmacy)]*

In such cases, pharmacists relied not only on the dispensing software but also on the staff responsible for labelling the prescription to alert them to any warnings. The panel members noted that miscommunications and failures to flag warnings were common in practice, often leading pharmacists overlooking warnings.

*“You would hope also in this, like I said before about staff flagging up, if they were new that something would be written on the prescription to say that”. [Participant 3, manager (large multiple chain)]*

Additionally, depending on healthcare professionals to have already verified certain sub-tasks can impact CPs' compliance. This situation was frequently encountered with prescriptions from secondary care, where a pharmacy was unlikely to possess the medical history of walk-in patients yet responsible for clinically validating the prescription.

*“If someone’s handing in a green prescription from the hospital, say, they wouldn’t have a list of the repeats. And then there’s always the temptation to say, well it’s come from the hospital, they must know what they’re doing so it’s fine”. [Participant 2, manager (Medium chain)]*

*“It could be that a GP could assume that the pharmacist will check that, and the pharmacist assumes the GP has already checked that, then that’s your Swiss cheese holes lining up, isn’t it. When really both should check it”. [Participant 3, manager (large multiple chain)]*

## Discussion

Our findings revealed that deviations from SOPs occur when CPs clinically check prescriptions. CPs seemed to exhibit a behaviour similar to that observed amongst doctors [23], where the application of discretion, judgment, and reliance on unwritten rules played a role in shaping their decisions, often leading to less compliance to protocols. Our findings resonate with the *varieties in human work* theory [24], wherein SOPs represent the prescribed work, referred to as *work-as-prescribed* (WAP), and pharmacists' checking represent real work, termed *work-as-done* (WAD). WAP is typically formulated by distant senior members or experts and are considered the safe and ideal way to perform tasks. WAD, on the other hand, represents real activities carried out to achieve specific goals in a particular context, making it challenging to prescribe in a set of protocols. These variations between the two aspects of work were evident in our data, with notable differences observed between the prescribed SOPs and practices of CPs.

Nonetheless, it is important to acknowledge that this study obtained work-as-done data through a simulation exercise, which is a limitation when compared to real-life observations, due to the artificial environment that limits typical workplace interferences [25, 26]. However, the simulated environment provided an opportunity to investigate deviations from SOPs by pharmacists, extending beyond typical workplace conditions. This challenges the prevailing notion that deviations primarily stem from environmental factors imposed by work conditions [6, 11].

Most significantly, our research highlights the existence of deviations from SOPs even in controlled environments. This finding underscores the enduring influence of the aforementioned factors on pharmacists' daily practices, suggesting that despite variations in work conditions, deviating from SOPs might be featured regularly in their practice. Our panel members partially attributed such deviations to unrealistic SOPs expectations hindering compliance, occasionally serving as protective measures for organisations but being impractical. Furthermore, they pointed out that pharmacists often adapt their approach, focusing only on the sub-tasks they consider relevant, akin to consultants’ anaesthetists consulting emergency segments of protocols rather than following entire checklists, as observed by Phipps et al., [27].

### Proposed reasons for deviating from SOPs

One primary reason for deviating from SOPs was suggested to be pharmacists’ tendency to prioritise professional judgment, consistent with Thomas et al.’s [6] observation. Professional judgement is thought to introduce a degree of flexibility when following SOPs within healthcare; this is due to the dynamic nature of healthcare, where protocols may not always align with the needs of individual patients [8, 28, 29]. In Jones et al.’s [10] study of CPs, for example, their interviewees felt that professional judgment sometimes led them to deviate from SOPs in order to enact a more suitable action for patients. However, the exercise of professional judgement within community pharmacy remains somewhat unclear, emphasising the necessity for additional research into how CPs apply professional judgement. This is especially significant in the context of community pharmacy, where professional judgment is shaped by professional, commercial, and personal factors. Conflict, in such settings, arises due to the retail environment, the complex remuneration structure and high workload, which may not necessarily help in prioritising patient care [30].

Our findings emphasise the reliance of pharmacists on presumptions regarding the completion of specific sub-tasks by other healthcare professionals (HCPs), during prescription generation. Such reliance extends further to relying on staff members to highlight warnings from dispensing software. This challenge is exacerbated by documented instances of deficient communication within community settings [31], thus undermining the intended efficacy of dispensing software designed to assist pharmacists in detecting inappropriate prescribing. The focus group discussions revealed pharmacists occasionally assume issues have been addressed by the prescribing HCP or flagged by dispensers, resulting in occasional lapses in their examination of those sub-tasks. This dimension of reliance on other individuals/systems further complicates the checking task, particularly given prior research [14, 32] indicating that CPs already incorporate a degree of guess-work in their practice. The panel members noted such reliance is common in practice, particularly among new, one-off patients and/or those receiving care in secondary care; where poor information exchange is likely [33].

Also, the perception of patients' preferred outcomes by CPs, with an emphasis on efficiency as a priority for patients, was suggested as a potential contributor to deviations. This finding aligns with previous research [11, 15] showing pharmacists frequently making trade-offs to meet patients' expectations. However, patients' preference for efficiency in pharmacy is rather assumed, as there is a lack of evidence from their perspective to support this inclination. Here we reveal an interplay between safety considerations and assumed patient expectation for operational efficiency that influences pharmacists' decision-making. Consequently, pharmacists may opt for less comprehensive checks to promote efficiency; an example of that is observed with walk-in patients where the absence of patient history may hinder meeting the minimum SOP requirements. Moreover, as with previous studies [11, 31], work conditions were identified as a hindrance to following SOPs. One participant argued that CPs’ prolonged period of operation in high-demand environments has gradually shaped their practices, leading to a tendency to conduct fewer checks. This sheds light on the deviations observed in our study, where pharmacists had ample time and no interruptions but still chose to check what they deemed relevant.

### Strengths and limitations

This study's strengths include its diverse sample, which closely mirrors the real-world pharmacist population, and the controlled environment that minimises external factors like interruptions and time pressure. However, the relatively small sample size (12 CPs), while diverse, may constrain the generalisability of the findings to a broader pharmacists’ population, notably with the under-representation of highly experienced pharmacists. Also, the controlled research setting, while advantageous for minimising external influences and reducing the risk of confounding variables, may not fully replicate the dynamic and high-pressure conditions encountered in practice.

The use of think-aloud provided valuable insights into pharmacists' decision-making. Nevertheless, it is essential to acknowledge that tacit knowledge may have influenced CPs checking, which might not have been verbalised during the think-aloud process—an acknowledged limitation of this technique [34]. To mitigate this, participants were prompted, when needed, to verbalise each aspect they checked. Furthermore, almost all deviations carry the potential for harm ranging from insignificant to severe. However, we acknowledge that this may not always manifest in practice. In this study, we ensured uniform importance across all sub-tasks. It is important to note, however, that the scope of this study may be limited to CPs practicing in England.

### Implications

This study unveiled a tendency among CPs of omitting aspects of SOPs, calling for a reconsideration of the significance and repercussions of these protocols. By uncovering this aspect, our study underlines the need for a more nuanced approach to addressing protocol compliance within the pharmacy profession, acknowledging that these deviations might be rooted in longstanding habits. The complexities associated with clinical checking [13, 15], which heavily depend on clinical reasoning, offer partial insights into the observed deviations. However, an investigation into the formation of pharmacists' clinical judgment is warranted. Furthermore, we advocate for reforms in SOPs to strike a balance between compliance and flexibility, especially in tasks requiring clinical judgment.

## Conclusion

This study suggests the presence of deviations from established protocols during the clinical checking of prescriptions within community pharmacies. Such deviations were observed under uninterrupted, controlled conditions, diverging from the typical workplace environment. This observation indicates a pattern among pharmacists wherein strict adherence to SOPs may not consistently be followed, regardless of environmental factors. The identified contributing factors to such practice encompass the exercise of professional judgment, reliance on others, and the prioritisation of patient preferences, particularly concerning efficiency.

Future work should investigate how the risks associated with deviation from clinical checking SOPs can be managed and explore strategies to strike a balance between efficiency and protocol compliance. This balance aims to maintain efficiency while mitigating potential risks associated with noncompliance to established procedures.

## Supplementary Information

Below is the link to the electronic supplementary material.

Supplementary file1 (PDF 304 KB)
