# Supplementary material for: Protocols versus practice: unravelling clinical checking variations in community pharmacies in England—a multi-method study
Source: Int J Clin Pharm. 2024 Jun 1;46(5):1114–23. doi: 10.1007/s11096-024-01743-9 (PMC11399187; doi:10.1007/s11096-024-01743-9)
Supplement: Supplementary file 1 — Supplementary file1 (PDF 304 KB) [file 11096_2024_1743_MOESM1_ESM.pdf]

Virtual prescriptions (Listed from 1-3 consecutively)

Pharmacy Stamp

Age

65 Years

D.o.B

17/02/1956

Title Forename Surname & Address

Mr John Macaulay
52 Broadmoor Road
Manchester
M20 3WF

Please don't stamp over age box

No. of days' treatment
NB. Ensure dose is stated

NHS Number

Endorsements

Naproxen 500mg tablets
Take ONE tablet BD
Quantity: 56 tablets

Paracetamol 500mg tablets
Take ONE or TWO tablets QDS PRN
Quantity: 100 tablets

Signature of Prescriber

J. Parker

Date

16/04/2022

For dispenser
No. of
Prescripns
on form

Dr J Parker - **234567**
13 Beaufort Street
Manchester
M20 6QN
Tel: 0101 654321

NHS

FP10SS060

PRINTED SERIAL NUMBER

Mr John Macaulay
52 Broadmoor Road
Manchester
M20 3WF

Next review due: 20/11/2022
Last issued: 20/11/2021

------------ medication information -----------

Citalopram 10mg tablets – ONE tablet to be taken DAILY, 28 tablets

Cavilon Durable barrier Cream – As directed, 368 grams

Hypromellose 0.3% - APPLY as required, 1 x 10mls

Metformin 500mg tablets - ONE tablet to be taken THREE times DAILY, 84 tablets

Sitagliptin 100mg Tablets - ONE tablet to be taken DAILY, 28 tablets

Pharmacy Stamp

Age

39 Years

D.o.B

28/02/1982

Title Forename Surname & Address

Mrs Emma Kimberling
52 Moseley Avenue
Manchester
M19 3BW

Please don't stamp over age box

No. of days' treatment
NB. Ensure dose is stated

NHS Number

Endorsements

Labetalol 200mg tablets
Take ONE tablet BD
Quantity: 56 tablets

Aspirin 75 mg tablets
Take TWO tablets OD
Quantity: 56 tablets

Signature of Prescriber

J. Parker

Date

16/04/2022

For dispenser
No. of
Prescripns
on form

Dr J Parker - **234567**
13 Beaufort Street
Manchester
M20 6QN
Tel: 0101 654321

NHS

FP10SS060

PRINTED SERIAL NUMBER

Mrs Emma Kimberling
52 Moseley Avenue
Manchester
M19 3BW

Next review due: 20/11/2022
Last issued: 20/11/2021

------------ medication information -----------

Folic acid 400mcg tablets – ONE tablet to be taken DAILY, 28 tablets

Paracetamol 500mg tablets – ONE or TWO tablets to be taken FOUR times DAILY when required, 224 tablets

Sertraline 50mg tablets – ONE tablet to be taken DAILY, 28 tablets

Pharmacy Stamp

Age

5 years

D.o.B

28/02/2016

Please don't stamp over age box

Title Forename Surname & Address

Mr Hammam Hussein
52 Humber Road
Manchester
M17 3WF

No. of days' treatment
NB. Ensure dose is stated

NHS Number

Endorsements

Amoxicillin 500mg capsules
Take ONE capsule TDS for five days
Quantity: 15 capsules

Soluble prednisolone 5mg tablets
Take Four tablets (20mg) once every morning for Three days
Quantity: 12 Tablets

Aerochamber Plus with Mask
Quantity: 1 device

Signature of Prescriber

J. Parker

Date

16/04/2022

For dispenser
No. of Prescripns on form

Dr J Parker - **234567**
13 Beaufort Street
Manchester
M20 6QN
Tel: 0101 654321

NHS

FP10SS060

PRINTED SERIAL NUMBER

Mr Hammam Hussein
52 Humber Road
Manchester
M17 3WF

Next review due: 20/11/2022
Last issued: 20/11/2021

------------ medication information -----------

Clenil Modulite 50micrograms/dose inhaler – TWO PUFFS TO BE TAKEN TWICE DAILY, 1 x 200 dose.

Diprobase Cream – apply liberally as directed, 500g

Salbutamol 100micrograms/dose inhaler CFC free _ Inhale ONE TO TWO PUFFS FOUR TIMES A DAY WHEN REQUIRED, 2 X 120 dose.
